# Temporal patterns of endophytic microbial heterogeneity across distinct ecological compartments within the *Panax ginseng* root system following deforestation for cultivation

**DOI:** 10.3389/fmicb.2024.1402921

**Published:** 2024-05-02

**Authors:** Zhenting Shi, Limin Yang, Meiling Yang, Kexin Li, Li Yang, Mei Han

**Affiliations:** Cultivation Base of State Key Laboratory for Ecological Restoration and Ecosystem Management, College of Traditional Chinese Medicine, Jilin Agricultural University, Changchun, China

**Keywords:** *Panax ginseng*, ecological compartment, endophyte, microbiome, diversity

## Abstract

Alterations in the microbial community significantly impact the yield and quality of ginseng. Yet, the dynamics of microbial community shifts within the root endophytes of ginseng across varying cultivation periods remain inadequately understood. This study zeroes in on the microbial community variations within the xylem (M), phloem (R), and fibrous roots (X) of ginseng during the fourth (F4) and fifth (F5) years of cultivation, aiming to bridge this research gap. We assessed soil physicochemical properties, enzyme activities, and nine individual saponins, complemented by high-throughput sequencing techniques (16S rDNA and ITS) to determine their profiles. The results showed that cultivation years mainly affected the microbial diversity of endophytic bacteria in ginseng fibrous roots compartment: the ASVs number and α-diversity Chao1 index of bacteria and fungi in F5X compartment with higher cultivation years were significantly higher than those in F4X compartment with lower cultivation years. It is speculated that the changes of fibrous roots bacterial groups may be related to the regulation of amino acid metabolic pathway. Such as D-glutamine and D-glutamate metabolism D-glutamine, cysteine and methionine metabolism regulation. The dominant bacteria in ginseng root are Proteobacteria (relative abundance 52.07–80.35%), Cyanobacteria (1.97–42.52%) and Bacteroidota (1.11–5.08%). Firmicutes (1.28–3.76%). There were two dominant phyla: Ascomycota (60.10–93.71%) and Basidiomycota (2.25–30.57%). Endophytic fungi were more closely related to soil physicochemical properties and enzyme activities. AN, TK, OP, SWC and EC were the main driving factors of endophytic flora of ginseng root. *Tetracladium* decreased with the increase of cultivation years, and the decrease was more significant in phloem (F4R: 33.36%, F5R: 16.48%). The relative abundance of *Bradyrhizobium, Agrobacterium* and *Bacillus* in each ecological niche increased with the increase of cultivation years. The relative abundance of *Bradyrhizobium* and *Agrobacterium* in F5X increased by 8.35 and 9.29 times, respectively, and *Bacillus* in F5M increased by 5.57 times. We found a variety of potential beneficial bacteria and pathogen antagonists related to ginseng biomass and saponins, such as *Bradyrhizobium*, *Agrobacterium*, *Bacillus* and *Exophiala*, which have good potential for practical application and development.

## 1 Introduction

Endophytes are microorganisms designated to reside in the living internal tissues of plants without causing any immediate, obvious symptoms of disease (Bacon and White, 2000). They can co-evolve with plants and can directly or indirectly promote plant growth and resist harmful microorganisms (Wu et al., 2021). In medicinal plants, endophytes can also promote the production and accumulation of secondary metabolites in medicinal plants (Huang et al., 2018; Wu et al., 2021). In 1993, scientists isolated *Taxomyces andreanae* (Stierle et al., 1993), an endophytic bacterium capable of producing paclitaxel from *Taxus chinensis* (Pilger) Rehd., which set off a boom in the study of endogenous functional strains of medicinal plants and provided a new way for the development of new drugs for microbial resources and the protection of medicinal plants. Therefore, it is important to study the dynamics of endophytes in medicinal plants at both time and space scales to grasp the role and rule of microorganisms in the accumulation of secondary metabolites in medicinal plants (White et al., 2019).

The study of endophytic bacteria is not only beneficial to solve the problem of disease resistance and yield increase, but also lays a foundation for breaking the barrier of continuous cropping. More interestingly, these endophytic bacteria can also be directly involved in the production and biochemical transformation of active ingredients in medicinal plants, for example, *Bacillus subtilis* KX230132.1 can act as an effective stimulant to increase the concentration of ginsenosides in ginseng (Song et al., 2017). The endophytic fungus *Phoma* sp. May produce ginsenoside Re, while *Burkholderia* sp. GE 17-7 can convert ginsenoside Rb_1_ to ginsenoside Rg_3_ (Fu et al., 2017). The rare ginseng saponins Rg_3_ has a low content in plants and has very good anti-tumor activity, and the study of endophytic bacteria is also of great significance for the development of ginsenosides (Peng et al., 2021; Oh et al., 2022).

Endophytes are distributed in different ecological compartments of plants, such as xylem, phloem, stem and leaf, etc. Endophytes in these ecological compartments play their unique functions in the growth and fibrous root, development of plants (Berendsen et al., 2012; Schlaeppi and Bulgarelli, 2015; Rodríguez et al., 2020; Trivedi et al., 2020). However, the current research on endophytes is still in the initial stage, and most of them focus on the apparent characteristics of endophytes on growth and development, effective components and antibacterial activity of ginseng (Hou et al., 2012; Quan et al., 2013; Wang et al., 2015; Park et al., 2017; Song et al., 2017; Wei et al., 2021). The distribution law of endophytes in different ecological compartments and the mechanism of action with the change of cultivation years are still unclear. Especially in ginseng, a medicinal plant with high economic value, the study of endophytic flora in different ecological compartments along with cultivation years is still blank, which constitutes a major obstacle to understanding the interaction between endophyte and host plant.

Ginseng stands as the foremost medicinal material, boasting extensive applications across pharmaceutical, food, and cosmetic industries. The environment of deforest-grown ginseng closely mirrors the natural conditions favorable for its growth. The root endophytic flora, in particular, may significantly influence the growth and accumulation of medicinal components in ginseng. This presents a crucial area for exploring innovative growth promotion and disease control methods. Studying the effects of cultivation duration on the endophytic flora within different ecological compartments of the root system is therefore of paramount importance. Such research not only addresses the challenges of disease resistance and yield improvement for ginseng but also establishes a foundation for the sustainable development of resources. It holds significant potential for the advancement of ginsenosides and their biotransformation, making it a research avenue worthy of further exploration. In our study, we sequenced the bacterial and fungal communities within the xylem, phloem, and rhizomes of 4-year-old and 5-year-old ginseng plants grown in the same deforest soil environment. We also quantified the biomass in each ecological compartment of the above- and underground parts. Moreover, we assessed the concentrations of nine monomeric saponins, nine physicochemical properties, and eight enzyme activities in the rhizosphere soil of each compartment. Using Spearman’s correlation coefficient, we analyzed the relationships between endophytes and the physicochemical properties, enzyme activities, monomeric saponins, and biomass to explore the co-evolutionary dynamics among endophytes, ginseng, and soil. Our hypotheses include: (1) The endophytes in each ecological compartment might have undergone varying degrees of change with increased cultivation time; (2) There might be a significant correlation between endophytes and the quality and yield of ginseng. This study aims to elucidate the interactions between endophytes, ginseng, and soil, providing a scientific foundation for the cultivation and application of ginseng.

## 2 Materials and methods

### 2.1 Field experiments and sample collection

Samples were collected from the ginseng main production area located in Yanbian County, Jilin Province, China (N42°22′36.74″E127°06′37.59″ see Supplementary Figure 1 for details). The soil type was deforest black loam. The region has a temperate continental monsoon climate with an average annual rainfall of 574.9 mm. On 17 September 2021, we collected samples of 4-year-old (3-year-old seedlings transplanted for one year; F4) and 5-year-old (3-year-old seedlings transplanted for two years; F5) ginseng, respectively. A randomized grouping design was used, with six biological replicates of samples from each cultivation year, and a total of 12 plots of 20 m^2^ each. The collection followed a five-point sampling method and ensured that over 20 healthy ginsengs were selected for each replicate, amounting to over 250 ginsengs and soil samples.

### 2.2 Sample handling

All sampling instruments were sterilized in advance. Using the five-point sampling method, the topsoil of 1–2 nm was removed to harvest the ginseng. The collected samples, including stems, leaves, ginseng roots and rhizosphere soil, were then sorted, labeled, and placed in sampling boxes equipped with ice packs. These were stored under low temperatures sampling box and transported back to the laboratory for prompt sample processing. After cleaning, measurements of stem length, stem diameter, fresh stem weight, and leaf count were taken. The dry weight was recorded after drying the samples at 60°C in constant temperature drying oven. To clear the rhizosphere soil within a 2 mm radius around the ginseng root, a brush was used (Huang et al., 2022), followed by natural air drying for the assessment of physicochemical properties. The ginseng roots were cleansed with sterile water to remove surface debris. They were then disinfected by soaking in 75% alcohol for 30 s and in 2.5% sodium hypochlorite for 10 min, with a drop of Tween 80 added to every 100 ml of 2.5% sodium hypochlorite solution. The roots were rinsed with sterile water two to three times to completely remove the disinfectant. The effectiveness of the surface sterilization was verified by applying the last rinse water onto a petri dish to check for microbial growth. Finally, the roots were dried with sterile filter paper, fibrous root (X) and xylem (M) and phloem (R) from the middle part of the ginseng were extracted under sterile conditions (Gao et al., 2021). These were stored at −80°C for DNA extraction and subsequent 16S rDNA and ITS analysis. The biomass of each compartment was calculated, including: xylem fresh weight (MRW), phloem fresh weight (RFW), branch root fresh weight (BRFW), fibrous root fresh weight (XFW), stem fresh weight (SFW), xylem dry weight (MDW), phloem dry weight (RDW), branch root dry weight (BRDW), fibrous root dry weight (XDW), stem dry weight (SDW).

### 2.3 Soil physical and chemical properties and enzyme activity tests

We tested the physical and chemical properties of the soil by estimating the soil water content (SWC), pH, electrical conductivity (EC), soil organic matter (OM), total nitrogen (TN), total phosphorus (TP), total potassium (TK), available nitrogen (AN), and available phosphorus (OP). The detection of these indices was carried out by the drying method, the potentiometric method, the electrode method, the direct heating method, the automatic nitrogen fixation method, the sodium hydroxide alkali-soluble molybdenum antimony colorimetric method, the sodium hydroxide melting method, the alkali dissolution diffusion method, and the UV/visible spectrophotometer method, respectively (Shi-dan, 2000). The enzyme activities, including soil cellulase (S-CL), soil β-glucosidase (S-β-GC), acid protease (S-AcPr), soil urease (S-UE), soil acid phosphatase (S-ACP), soil sucrase (S-SC), soil catalase (S-CAT), and soil dehydrogenase (S-DHA), were all measured using kits supplied by Solarbio (Beijing, China). The contents of nine monomeric saponins Rg_1_, Re, Rf, Rb_1_, Rc, Rh_1_, Rb_2_, Rb_3_ and Rd were determined by high performance liquid chromatography method of Zhang (2019) was adopted.

### 2.4 DNA extraction, PCR amplification, and high-throughput sequencing

DNA from each sample was extracted using a commercial kit, with nuclease-free water serving as the blank control. The extracted total DNA was eluted in 50 μL of elution buffer and stored at −80°C for subsequent PCR amplification. The sequences were amplified in two rounds using the reaction conditions and primers listed in Supplementary Tables 1–10, employing nested PCR for endophytic 16S rDNA as described by Ikenaga and Sakai (2014). Primers for endophyte 16S rDNA and two rounds of amplification for endophyte ITS were detailed in Supplementary Tables 1–10, respectively, as per Yao et al. (2019). PCR products were verified through 2% agarose gel electrophoresis. Throughout the DNA extraction process, ultrapure water was used instead of sample solution as a negative control to eliminate the possibility of false-positive PCR results. The PCR products were purified using AMPure XT beads (Beckman Coulter Genomics, Danvers, MA, USA) and quantified with Qubit (Invitrogen, USA). The quality of the purified PCR products was assessed using the Agilent 2100 Bioanalyzer (Agilent, USA) and Illumina library quantification kit (Kapa Biosciences, Woburn, MA, USA). Sequencing was performed on the NovaSeq 6000 system with 2 × 250 bp paired-end sequencing using the NovaSeq 6000 SP Reagent Kit (500 cycles).

### 2.5 Data analysis

Sequencing was conducted on the Illumina NovaSeq platform by LC-Bio, China. Paired-end reads were demultiplexed based on unique barcodes, with barcode and primer sequences removed. The FLASH tool was utilized for merging paired reads. For raw data processing, fqtrim (v0.94) was employed for quality filtering to ensure high-quality reads. Chimeric sequences were filtered out using Vsearch software (v2.3.4). The relative and characteristic abundances of bacteria and fungi in each sample were normalized using the SILVA (release 138) classifier, the RDP database, and the UNITE database.

Based on the amplicon sequence variation (ASV) feature sequences and abundance tables, we conducted analyses of α and β diversity. We evaluated microbial richness and diversity using the Chao1 and Shannon diversity indices. To analyze the differences between groups, we employed the Kruskal–Wallis test. The Bray-Curtis dissimilarities of the ASV community matrix were visualized in two dimensions using non-metric multidimensional scaling (NMDS). A stacked chart was utilized to classify the relative abundances of the top 30 species, showcasing the distinct relative abundances for each sample. Compartment-specific biomarkers were identified through variance analysis using LEfSe. The Spearman correlation coefficient was applied to analyze the relationships between soil chemical properties, enzyme activities, and the microbial relative abundance in RS and BS across various tillage years, with the results presented in a cluster heat map. Function prediction based on PICRUSt2 utilized the KEGG database for functional annotation. Data visualizations include the ASV petal plot, alpha diversity and violin plots, stacked plots, LEfSe ANOVA, relative abundance clustering heat maps, and STAMP ANOVA plots. The analyses were conducted using the “plotrix,” “ggplot2 (3.2.0),” vegan, nsegata-lefse, and corrplot packages in R version 3.4.4. All other graphics were created with the R package (version 3.5.2 “Easy Amplicon”). The study data has been uploaded to NCBI under the accession number PRJNA100474.

## 3 Results

### 3.1 Analysis of microbial composition and diversity of endophytic communities

**FIGURE 1:**
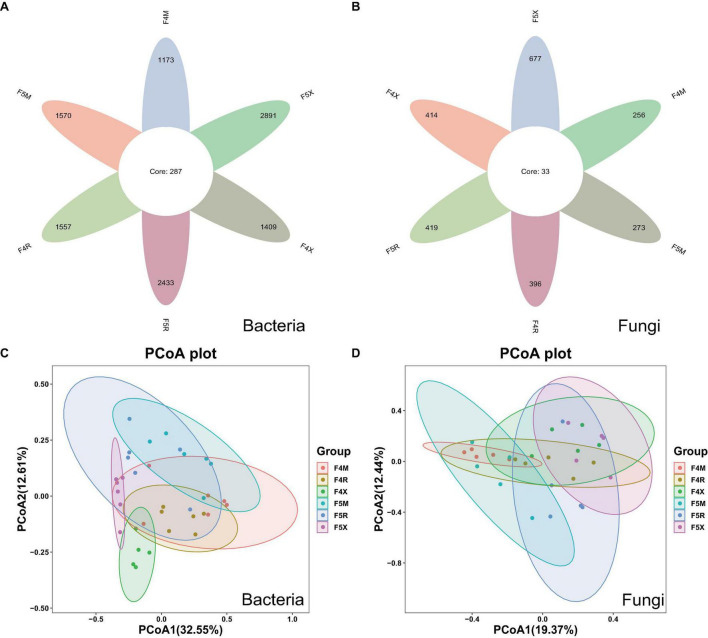
Changes in the number of ASVs and Beta–diversity in all groups of endophyte samples. The petal diagram **(A)** shows bacteria and **(B)** shows fungi, NMDS showed the structure of the bacteria **(C)** and fungal **(D)** community among different planting years of ecological compartments in ginseng root endophyte.

The sequences for bacteria and fungi were 2,761,817 and 2,429,270, respectively. In total, 6,259 bacterial ASVs and 1,635 fungal ASVs were detected, with 287 bacterial ASVs and 33 fungal ASVs shared across samples. Unique bacterial ASVs identified in various ecological compartments—F4M, F5M, F4R, F5R, F4X, and F5X—were 1,173, 1,570, 1,557, 2,433, 1,409, and 2,891, respectively, while the numbers of unique fungal ASVs were 256, 273, 396, 419, 414, and 677, respectively (Figures 1A, B). The quantity of bacterial ASVs in each compartment increased with the years of cultivation, showing increases of 33.84% in the xylem, 56.26% in the phloem, and 105.18% in the fibrous roots (Figure 1A). Similarly, the count of fungal ASVs in all compartments increased with cultivation duration, with increments of 6.64% in the xylem, 5.80% in the phloem, and 63.52% in the fibrous roots (Figure 1B).

Principal coordinate analysis (PCoA) accounted for 45.16% of the variability, highlighting significant distinctions between F4X and F5X and other groups, notably showcasing their significant separation. This suggests that fibrous roots exhibit distinct differences from other ecological compartments, with these differences becoming more pronounced over different years of cultivation and increasingly distinct from the xylem and phloem as the years progress (Figure 1C). PCoA also explained 40.81% of the variation, demonstrating that similarities within the same compartments across different years were higher than those across different compartments within the same year of planting. The overlaps between F4M and F5M, F4R and F5R, and F4X and F5X suggest minor differences within these groupings. The separation between M and X indicates a larger disparity, which becomes more pronounced with increasing years of cultivation, suggesting that the differences between the xylem and fibrous roots grow as the cultivation period extends (Figure 1D).

**FIGURE 2:**
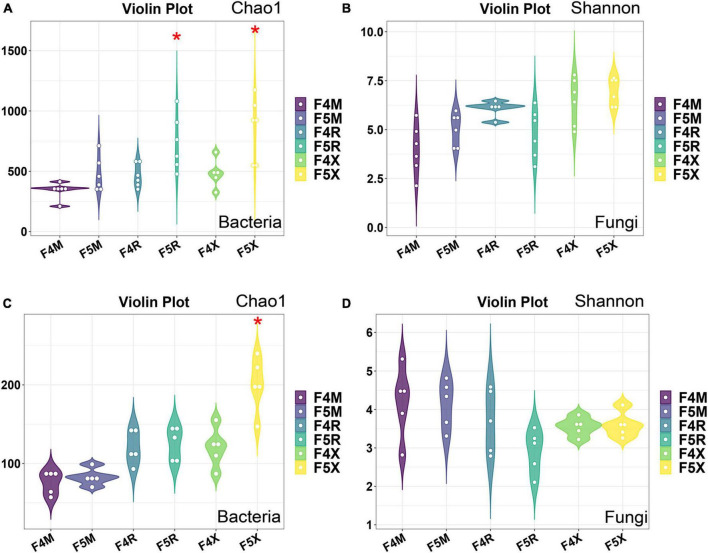
Alpha-diversity of microorganisms in ginseng at different ecological compartments and years of cultivation. Chao 1 for bacteria **(A)**, Shannon for bacteria **(B)**, Chao 1 for fungi **(C)**, and Shannon for fungi **(D)**. Data are presented as the mean ± standard error (*P < 0.05, according to Kruskal–Wallis test).

In this study, in order to evaluate the richness of species, we chose Chao1 index; To assess diversity, we chose the Shannon index. For the α-diversity Chao1 index of bacteria, F5R is significantly higher than F4R and F5X is significantly higher than F4X (Figure 2A). The Chao index F5X of fungi was significantly higher than that of F4X (Figure 2C), while the Shannon index of bacteria and fungi in different ecological compartments had no significant difference between different cultivation years (Figures 2B, D).

### 3.2 Composition of the root microbial community at phylum and genus level

Based on the ASV feature sequences obtained, we selected the top 30 abundant species for detailed classification at the phylum and genus levels, with those species exhibiting a relative abundance greater than 1% categorized as dominant bacteria.

**FIGURE 3:**
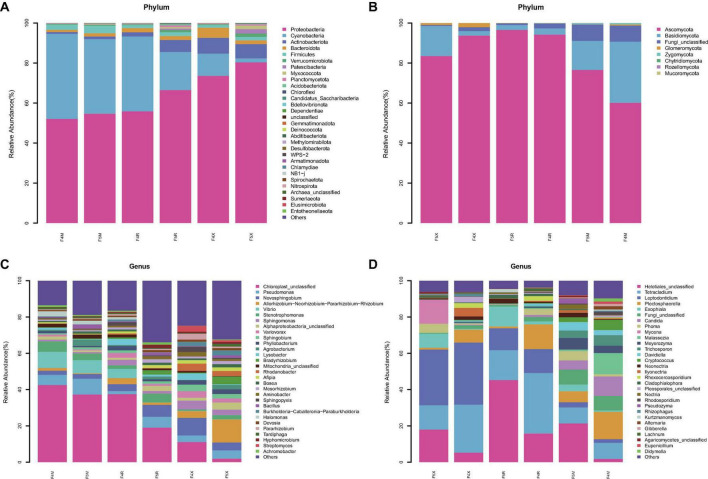
Species abundance stacked bar chart (TOP30). Average relative abundances change of bacterial phylum **(A)** and fungal phylum **(B)**, bacterial genus **(C)** and fungal genus **(D)**.

The dominant bacterial phyla include four groups: Proteobacteria, with a relative abundance ranging from 52.07 to 80.35%; Cyanobacteria, ranging from 1.97 to 42.52%; Bacteroidota, ranging from 1.11 to 5.08%; and Firmicutes, ranging from 1.28 to 3.76%. Notably, the relative abundance of Cyanobacteria decreased gradually with age across all compartments (F4M: 42.52%, F5M: 37.28%, F4R: 37.47%, F5R: 19.02%, F4X: 11.11%, F5X: 1.97%). Bacteroidota and Actinobacteriota showed a decreasing trend in the fibrous roots with age, while most others, such as Proteobacteria, increased in each compartment with the increase of cultivation years (F4M: 52.07%, F5M: 54.67%, F4R: 55.82%, F5R: 66.49%, F4X: 73.59%, F5X: 80.35%) (Figure 3A).

Two dominant fungal phyla were identified: Ascomycota, with relative abundances between 60.10 and 93.71%, and Basidiomycota, ranging from 2.25 to 30.57%. Ascomycota’s relative abundance in F5X decreased from 93.71 to 83.49% with age, while in F5M and F5R, it increased from 94.21% and 60.10% to 96.54% and 76.53%, respectively. Basidiomycota showed an opposite trend to Ascomycota, increasing in fibrous roots from 2.25 to 15.04% with age, while decreasing in phloem and xylem from 3.07% and 30.57% to 2.36% and 14.49%, respectively (Figure 3B).

Dominant bacterial genera included *Pseudomonas* (ranging from 1.71 to 8.69%) and *Novosphingobium* (ranging from 2.21 to 9.74%). The relative abundances of the genera *Allorhizobium*, *Neorhizobium*, *Pararhizobium*, and *Rhizobium* significantly increased in the F5X with the increase of planting years, whereas their relative abundances decreased in F5M and F5R. *Pseudomonas* had higher relative abundances in F5M and F5X (8.69 and 4.55%, respectively) compared to F4M and F4X (5.66 and 3.59%). *Stenotrophomonas* was less abundant in F5M (3.68%) than in F4M (5.81%), but had higher relative abundances in F5R (4.84%) and F5X (2.33%) compared to F4R (2.01%) and F4X (0.78%). *Bradyrhizobium*, *Agrobacterium*, and *Bacillus*—associated with biomass—showed increasing trends in relative abundance across all ecological compartments with cultivation years. *Bradyrhizobium* and *Agrobacterium* increased 8.35 and 9.29 times in F5X, respectively, and *Bacillus* increased 5.57 times in F5M (Figure 3C).

Dominant fungal genera included Helotiales_unclassified (ranging from 1.79 to 21.35%), *Tetracladium* (ranging from 8.77 to 33.36%), and *Leptodontidium* (ranging from 2.03 to 30.71%). Helotiales_unclassified showed a significant increase with cultivation years, highly correlated with biomass (F4M: 1.79%, F5M: 21.35%, F4R: 15.78%, F5R: 45.25%, F4X: 5.24%, F5X: 17.96%). *Exophiala* also increased with cultivation years, significantly enriched in F5X and correlated with biomass (F4M: 0.69%, F5M: 3.71%, F4R: 1.62%, F5R: 10.97%, F4X: 0.46%, F5X: 7.71%). *Tetracladium* and *Plectosphaerella* both showed a decrease with the increase of cultivation years (F4M: 8.88%, F5M: 8.77%, F4R: 33.36%, F5R: 16.48%, F4X: 34.21%, F5X: 30.71% for *Tetracladium*; and F4M:15.07%, F5M: 6.04%, F4R: 13.63%, F5R: 0.98%, F4X: 7.12%, F5X: 1.01% for *Plectosphaerella*, Figure 3D).

### 3.3 Differences in endophyte microbial community

In this study, LefSe analysis was employed to investigate the notable patterns of enrichment for bacteria and fungi within the endophytic communities of ginseng roots. It was observed that, in comparison to ginseng roots from shorter cultivation periods, those from longer cultivation periods (F5R and F5X ecological compartments) showed significant bacterial enrichment. Conversely, fungi demonstrated significant enrichment in the ginseng root endophytes from shorter cultivation periods and the F5M compartment.

**FIGURE 4:**
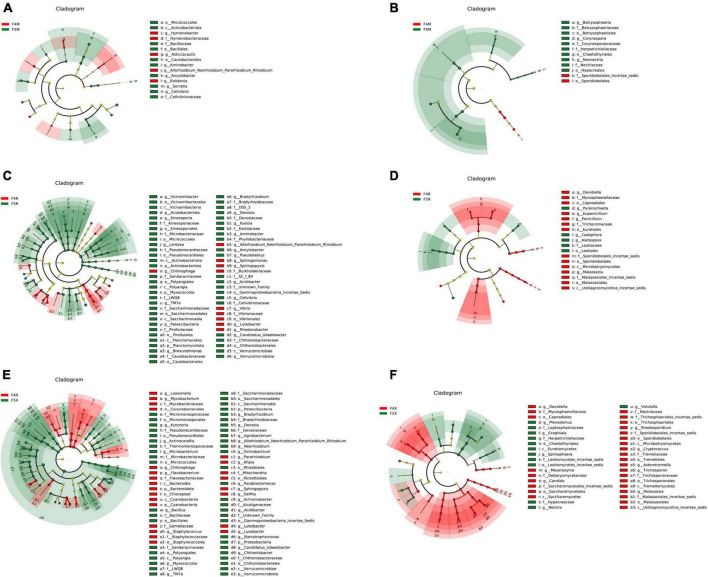
LEfSe analysis of among different planting years of ecological compartments in ginseng root endophyte. Arcograms of taxonomic evolutionary branches of two groups of significantly different bacteria **(A,C,E)** and fungi **(B,D,F)**, with different circle layers radiating from inside to outside representing the six taxonomic levels of the Phylum Orders Families and Genera, respectively, and each node representing a species classification at that level, with the greater the abundance of the species the greater the color range. The node color is yellow indicates that the species has no significant difference in the comparison group, the node color is red indicates that the species has significant difference species (i.e., biomarker) in the 4-year-old ginseng samples, and the node color is green indicates that the species has significant difference species (i.e., biomarker) in the 5-year-old ginseng samples, and the gates of the significant differences are directly labeled in the figure, and the other levels of the differences in species nodes were identified with letters (bacterial LDA > 3.5, fungal LDA > 3.0).

More specifically, the F4M compartment showed significant enrichment of the phylum Proteobacteria (including the genera *Ralstonia*, *Hymenobacter*, *Asticcacaulis*, and *Allorhizobium-Neorhizobium-Pararhizobium-Rhizobium*) and five bacterial species from the phylum Bacteroidota. The F5M compartment was notably enriched with ten bacterial species from the phyla Proteobacteria (including the genera *Ancylobacter* and *Aminobacter*), Firmicutes, and Actinobacteriota (Figure 4A).

In terms of fungi, the F4M compartment was significantly enriched with two species from the phylum Basidiomycota, and the F5M compartment was notably enriched with ten species from the phylum Ascomycota (including the genera *Neonectria*, *Botryosphaeria*, and *Corynespora*) (Figure 4B).

The F4R compartment exhibited significant enrichment of ten bacterial species from the phylum Proteobacteria (including the genera *Rhodanobacter*, *Sphingomonas*, *Sphingopyxis*, *Allorhizobium-Neorhizobium-Pararhizobium-Rhizobium*, *Vibrio*, and *Luteibacter*) and the genus *Chitinophaga* from the phylum Bacteroidota. The F5R compartment showed significant enrichment of fifty-three bacterial species from the phyla Proteobacteria (including the genera *Aminobacter*, *Lentzea*, *Pseudolabrys*, *Cellvibrio*, *Brevundimonas*, *TM7a*, *Devosia*, *Kaistia*, *Achromobacter*, and *Bradyrhizobium*), Acidobacteriota (including the genera *Vicinamibacter* and *Kineosporia*), Myxococcota, and Verrucomicrobiota (Figure 4C).

The F4R compartment was significantly enriched with fourteen fungal species from the phyla Ascomycota (including the genera *Davidiella*, *Eupenicillium*, and *Penicillium*) and Basidiomycota (*Malassezia*). The F5R compartment was notably enriched with five fungal species from the phylum Ascomycota (including the genera *Cadophora*, *Pyrenochaeta*, and *Alatospora*) (Figure 4D).

In the F4X compartment, significant enrichment was observed for twenty-four bacterial species from the phyla Firmicutes (*Staphylococcus*), Proteobacteria (including the genera *Pararhizobium*, *Sphingopyxis*, *Lysobacter*, *Afipia*, *Delftia*, and *Luteibacter*), Bacteroidota (*Chitinophaga*), Actinobacteriota (including the genera *Lawsonella* and *Mycobacterium*), and *Cyanobacteria*. In the F5X compartment, significant enrichment was seen for forty-six bacterial species from the phylum Proteobacteria (including the genera *Parablastomonas*, *Achromobacter*, *Stenotrophomonas*, *Ochrobactrum, Acidibacter*, *Agrobacterium*, *Allorhizobium-Neorhizobium-Pararhizobium-Rhizobium*, *Neorhizobium*, and *Bradyrhizobium*), the phyla Myxococcota and Verrucomicrobiota (including the genera *Candidatus Udaeobacter* and Chthoniobacter), Actinobacteriota (including the genera *Kutzneria*, *Actinocorallia*, and *Microbacterium*), Patescibacteria (*TM7a*), and Firmicutes (*Bacillus*) (Figure 4E).

The F4X compartment significantly enriched twenty-eight fungal species from the phyla Ascomycota (including the genera *Davidiella* and *Candida*) and Basidiomycota (including the genera *Cryptococcus*, *Asterotremella, Trichosporon*, *Malassezia*, *Rhodosporidium*, and *Meyerozyma*). The F5X compartment was notably enriched with twelve fungal species from the phylum Ascomycota (including the genera *Exophiala*, *Volutella*, *Plenodomus*, *Nectria*, and *Spirosphaera*) (Figure 4F).

### 3.4 Prediction analysis of functional genes of microbial metabolic pathways

The KEGG function prediction analysis performed using PICRUSt2 highlighted that the level 2 functional categories and their distribution across different cultivation periods within the F4 and F5 ecological compartments of ginseng root endophytes demonstrate considerable similarity, exhibiting either an increasing or decreasing trend from “M-R-X.”

**FIGURE 5:**
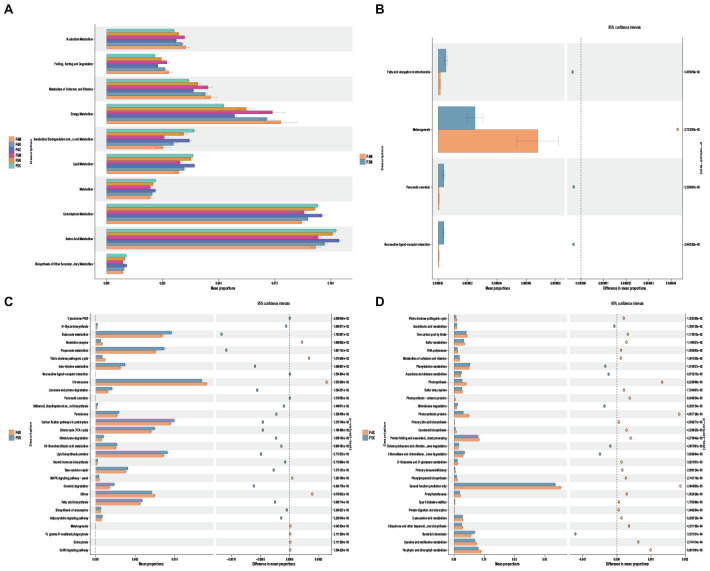
Potential functional roles of bacterial taxa across different ecological compartments in the root system. **(A)** Using PICRUSt2, we predicted bacterial community differences between the xylem(M) and phloem(R) and fibrous roots(X) in different ecological compartments and years of cultivation in the root system. **(B,C,D)** Xylem and phloem and fibrous roots in different years of cultivation at KEGG level 3. Differences with *p* < 0.05 are visualized using Stamp analysis, and significance was ascertained using Welch’s *t*-test.

Specifically, functions such as Nucleotide Metabolism, Folding, Sorting and Degradation, Metabolism of Cofactors and Vitamins, and Energy Metabolism gradually decreased from “M-R-X.” Conversely, other functions showed an increasing trend from “M-R-X,” including Xenobiotics Biodegradation and Metabolism, Lipid Metabolism, General Metabolism, Carbohydrate Metabolism, Amino Acid Metabolism, and Biosynthesis of Other Secondary Metabolites (Figure 5A).

Comparative analysis revealed minimal differences in functional categories between F4M and F5M, indicating a high level of stability in the functions of xylem endophytes (Figure 5B). Specifically, F5R was significantly enriched with functions related to Limonene and Pinene Degradation, Nitrotoluene Degradation, Geraniol Degradation, and Lipid Biosynthesis Proteins (Figure 5C). For F4X, there was significant enrichment in functions related to Sulfur Metabolism, Metabolism of Cofactors and Vitamins, Primary Bile Acid Biosynthesis, D-glutamine and D-glutamate metabolism, and cysteine and methionine metabolism. Meanwhile, F5X showed significant enrichment in Arachidonic Acid Metabolism, Phenylalanine Metabolism, and Ascorbate and Aldarate Metabolism (Figure 5D).

### 3.5 Correlation analysis between biomass, soil properties, saponin content, and microorganisms

The analysis utilizing Spearman correlation coefficients explored the relationships between endophytes and various physicochemical properties, enzyme activities, individual saponins, and biomass. This investigation aimed to delve into the co-evolutionary dynamics between ginseng endophytes and both ginseng itself and the soil environment. For this study, we conducted a correlation analysis between the top 5 most abundant bacterial and fungal phyla and nine individual saponins (Rg_1_, Rh_1_, Rf, Re, Rb_1_, Rb_3_, Rc, Rb_2_, Rd). Furthermore, we analyzed the correlations between the top 30 most abundant bacterial and fungal genera and nine soil physicochemical properties (SWC, pH, EC, OM, TN, TP, TK, AN, OP), eight enzyme activities (S-CL, S-β-GC, S-AcPr, S-UE, S-ACP, S-SC, S-CAT, S-DHA), and the biomass (MRW, RFW, BRFW, XFW, SFW, MDW, RDW, BRDW, XDW, SDW) and individual saponins within each ecological compartment (M, R, and X).

**FIGURE 6:**
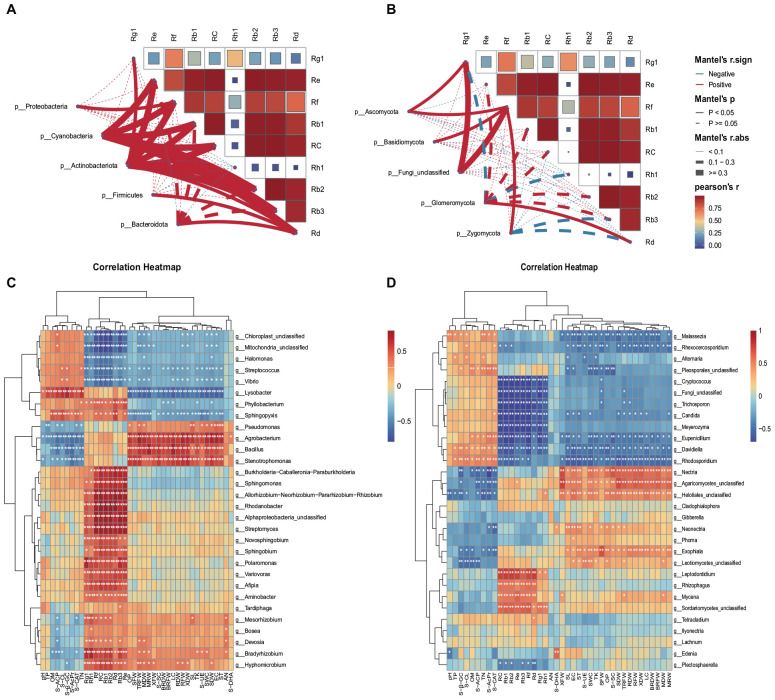
Spearman correlation coefficients. Spearman correlation coefficients between bacterial **(A)** and fungal **(B)** phylum communities and saponins. Spearman correlation coefficients between bacterial **(C)** and fungal **(D)** communities and environmental variables and saponins. The figure’s rows correspond to the top 30 species by abundance, and columns represent various environmental indicators. Significance levels are indicated as follows: *P < 0.05; **P < 0.01. Cells colored in red and blue signify positive and negative correlations, respectively. Soil water content (SWC), pH, electrical conductance (EC), soil organic matter (OM), total nitrogen (TN), total phosphorus (TP), total potassium (TK), available nitrogen (AN), and available phosphorus (OP), Soil cellulase (S-CL), Soil β-glucosidase (S-β-GC), Acid protease (S-AcPr), Soil Urease (S-UE), Soil acid phosphatase (S-ACP), Soil sucrase (S-SC), Soil catalase (S-CAT), Soil dehydrogenase (S-DHA), xylem fresh weight (MRW), phloem fresh weight (RFW), branch root fresh weight (BRFW), fibrous root fresh weight (XFW), stem fresh weight (SFW), xylem dry weight (MDW), phloem dry weight (RDW), branch root dry weight (BRDW), fibrous root dry weight (XDW), stem dry weight (SDW).

The findings indicate that endophytic bacteria demonstrated a stronger correlation with individual saponins compared to endophytic fungi. Bacteria, especially Cyanobacteria and Actinobacteriota, showed significant positive correlations with various saponins (Figure 6A), while fungi from the Ascomycota phylum exhibited significant positive correlations with Rg1, and Ascomycota, Basidiomycota, and Zygomycota showed positive correlations with Rf (Figure 6B).

Moreover, endophytic bacterial genera exhibited numerous positive correlations with individual saponins and ginseng biomass (Figure 6C). In contrast, endophytic fungal genera displayed fewer positive correlations and more negative correlations with individual saponins and ginseng biomass (Figure 6D). Bacteria mainly showed positive correlations with AN, OP, EC, TK, and SWC (Figure 6C) whereas fungi exhibited positive correlations with TP, OM, TN, AN, TK, OP, SWC, and EC (Figure 6D). The details are as follows:

(1) Among bacteria, *Agrobacterium*, *Stenotrophomonas*, *Bacillus*, and *Achromobacter* were significantly positively correlated with biomass across various parts of the ginseng plant. The genus *Bradyrhizobium* showed significant positive correlations with various biomass indicators such as TRFW, MDW, MRW, XDW, SFW, and SL. Unidentified Alphaproteobacteria demonstrated significant positive correlations with TRFW and several biomass measures. *Hyphomicrobium* was notably correlated with TRFW and various biomass metrics. *Pseudomonas* exhibited significant positive correlations with SL, ST, TRFW, MDW, and SDW. A variety of endophytic bacteria showed significant positive correlations with the nine individual saponins, including genera like *Sphingomonas*, *Novosphingobium*, *Bradyrhizobium*, *Streptomyces*, *Burkholderia-Caballeronia-Paraburkholderia*, *Allorhizobium-Neorhizobium-Pararhizobium-Rhizobium*, *Phyllobacterium*, *Sphingopyxis*, *Rhodanobacter*, and several others, highlighting their potential for novel discoveries (Figure 6C).

(2) In the realm of fungi, unclassified *Agaricomycetes*, *Helotiales*, *Exophiala*, and *Nectria* exhibited significant positive correlations with biomass across both aerial and subterranean parts of ginseng. *Neonectria* showed positive correlations with SL, ST, SDW, and TRFW. *Mycena* was positively correlated with XFW, MDW, and TRFW. *Leptodontidium*, *Rhizophagus*, *Mycena*, and unclassified Sordariomycetes demonstrated significant correlations with several individual saponins (Figure 6D), indicating a complex interplay between endophytes, ginseng biomass, and individual saponin content.

## 4 Discussion

### 4.1 The impact of cultivation duration on the diversity of endophytic microbes within ginseng roots across various ecological compartments

The cultivation duration predominantly impacts the microbial diversity within the fibrous roots compartment of ginseng. Our team’s prior research, along with studies by other scholars, has consistently shown a decrease in fungal diversity in ginseng soil with increasing cultivation years (Xiao, 2015; Xiao et al., 2016; Jin et al., 2022; Shi et al., 2024; Zhang et al., 2024). Furthermore, our recent investigations revealed that cultivation years exerted a more pronounced effect on the microbiome diversity within the fibrous root compartment. As the cultivation years advance, the number of bacterial amplicon sequence variants (ASVs) in each ecological compartment of ginseng roots tends to increase, with a notably greater expansion observed in bacterial populations. Particularly noteworthy is the significant elevation in the ASV numbers of bacteria and fungi, along with the α-diversity Chao1 index, within the F5X compartments characterized by longer cultivation periods, compared to the F4X compartments with shorter cultivation durations.

Moreover, our findings indicate a significant enrichment of bacterial groups in the F5R and F5X compartments compared to the F4R and F4X compartments (Figure 2). Additionally, the disparities in microbiomes between the xylem and fibrous roots become more pronounced with increasing cultivation years (Figures 1C, D). This suggests that bacterial populations may assume a dominant position within ginseng roots, particularly within the fibrous root compartment, as cultivation years progress.

### 4.2 The influence of cultivation duration on the community composition of endophytic microbes within ginseng roots in different ecological compartments

With increasing cultivation years, the potential disease resistance and potential saponin production synergistic ability of endophytic bacteria within the fibrous roots compartment decrease. Goodwin (2022) discovered that endophytes in ginseng seeds primarily comprised Proteobacteria, Firmicutes, Actinobacteria, and Bacteroidetes. Our study revealed that endophytic bacteria in ginseng roots mainly consisted of Proteobacteria, Firmicutes, Cyanobacteria, and Bacteroidota. Notably, the relative abundance of Actinobacteriota and Bacteroidota within the fibrous roots gradually decreased with age (Figure 2A). Actinomycetes, known for their broad sources, produce metabolites with high activity, including antibiotics, organic acids, steroids, and enzyme inhibitors, exhibiting various bioactive properties. Actinomycetes inhibit or eradicate plant diseases through mechanisms such as antibiosis, competition, and hyperparasitism (Chang et al., 2022; Goodwin, 2022). As cultivation years progress, the relative abundance of Proteobacteria and Firmicutes across all ecological compartments exhibits an increasing trend. Many Firmicutes members can produce spores resistant to dehydration and extreme conditions, serving as biological control agents against plant pathogens (Dong et al., 2018a,b). The prevalence of Proteobacteria may relate to its involvement in phosphate dissolution and indoleacetic acid, facilitating both early seed germination and subsequent plant growth (Vendan et al., 2010; Chowdhury et al., 2018; Goodwin, 2022). Overall, with cultivation duration extension, different beneficial bacteria manifest varied strategies of increase and decrease.

Li et al. (2023) highlighted Ascomycota as the dominant fungal phylum in ginseng roots. Our research expanded on this by including Basidiomycota in addition to Ascomycota. Ascomycota promotes plant resistance to pathogens and abiotic stresses (Dong et al., 2018a,b). We observed a decrease in Ascomycota abundance in X with increasing cultivation years, while it increased in R and M, correlating with planting duration (Figure 2C). Actinobacteriota exhibited significant positive correlations with many saponins (Figure 6A), while Ascomycota showed significant positive correlations with Rg1 and Rf (Figure 6B). The relative abundance of Actinobacteriota and Ascomycota in fibrous roots gradually decreased with age (Figure 2A). Our findings suggest that the decline in disease resistance and saponin production synergistic ability of endophytic bacteria is associated with prolonged cultivation years.

### 4.3 Co-evolution of ginseng root endophytic bacteria with soil physicochemical properties and biomass yield

Studies have indicated that soil pH, organic matter, and total nitrogen content are the most crucial and reliable predictors of bacterial and fungal community structure (Jin et al., 2022). Our research revealed that soil physicochemical properties and enzyme activities exhibited a stronger correlation with endophytic fungi (Figures 6C, D), with AN, TK, OP, SWC, and EC emerging as the primary driving factors.

Despite a decline in the content of some beneficial bacteria, our study demonstrated the enrichment of various advantageous endophytes associated with ginseng quality and yield across the ecological compartments of ginseng roots under long-term cultivation. *Tetracladium* showed a decrease with increasing cultivation years, with a more pronounced reduction observed in the phloem (F4R: 33.36%, F5R: 16.48%, Figure 3D). *Tetracladium*, known for its robust disease resistance and phosphorus-releasing properties, exhibits a mutual antagonistic relationship with root pathogens and positively correlates with crop yield, thereby exerting a beneficial impact on host health and growth (Hilton et al., 2021; Duan et al., 2022; Lazar et al., 2022). Conversely, *Exophiala* demonstrated an increase with prolonged cultivation (Figure 3D), significantly enriching in F5X (Figure 4F), and exhibiting a notable positive correlation with the biomass across all groups (Figure 6D). *Exophiala*, functioning not only as a functional degrading agent but also as a promoter of plant growth through hormone production and phosphorus uptake under abiotic stress conditions, underscores its significance (Xu et al., 2018).

The relative abundance of *Bradyrhizobium*, *Agrobacterium*, and *Bacillus*, significantly associated with biomass, exhibited an increasing trend across all ecological compartments with prolonged cultivation. Specifically, *Bradyrhizobium*, *Agrobacterium*, and *Bacillus* showcased remarkable increases, with *Agrobacterium* witnessing an 8.35-fold and 9.29-fold increment in F5X, and *Bacillus* experiencing a 5.57-fold increase in F5M, respectively (Figure 2B). Notably, *Agrobacterium* and *Bacillus* were notably enriched in F5X (Figure 4E), while both showed significant positive correlations with ginseng biomass across all parts (Figure 6C). *Bradyrhizobium*, renowned for its nitrogen-fixing ability and its role in providing nitrogen sources for plant growth and soil microorganisms, exhibited a significant positive correlation with nine monomeric saponins and biomass (TRFW, MDW, MRW, XDW, SFW, and SL) (Figure 6C). Moreover, studies have underscored *Bacillus*’s capacity to enhance the synthesis of ginseng rare ginsenoside CK active ingredients, while *Agrobacterium* promotes the synthesis of ginseng rare ginsenoside Rg3 and ginsenoside Rh2 active ingredients (Hou et al., 2012; Quan et al., 2013; Wang et al., 2015; Song et al., 2017). Additionally, *Leptodontidium* exhibited a significant positive correlation with several monomeric saponins (Figure 6D), indicating its role in stimulating plant growth through the release of volatile organic compounds (VOCs) (Berthelot et al., 2016).

These findings bolster the credibility of the correlations presented in this study, revealing numerous potential beneficial bacteria, pathogen antagonists, and microbial groups linked to ginseng biomass and saponins, thereby holding substantial promise for practical applications and further development.

## 5 Conclusion

In conclusion, our study elucidates the intricate dynamics of microbial communities within ginseng root under varying cultivation years. We identified key microbial taxa associated with ginseng quality, yield, and disease resistance, highlighting their potential as targets for enhancing ginseng cultivation practices. Furthermore, the correlations between soil physicochemical properties, enzyme activities, and microbial composition underscore the complex interplay between environmental factors and microbial ecology in ginseng cultivation. While our findings provide valuable insights, it is essential to recognize the limitations inherent in translating laboratory findings to real-world cultivation scenarios. Therefore, further validation and experimentation are warranted to fully leverage the practical application potential of these microbial discoveries in optimizing ginseng cultivation strategies.

## Data availability statement

The datasets presented in this study can be found in online repositories. The names of the repository/repositories and accession number(s) can be found below: https://www.ncbi.nlm.nih.gov/genbank/, PRJNA1004743.

## Author contributions

ZS: Writing – original draft, Writing – review & editing. MY: Writing – original draft. KL: Writing – original draft. LiY: Writing – review & editing. MH: Writing – review & editing. LimY: Writing – review & editing.
