# Supplementary material for: Temporal patterns of endophytic microbial heterogeneity across distinct ecological compartments within the Panax ginseng root system following deforestation for cultivation
Source: Front Microbiol. 2024 May 2;15:1402921. doi: 10.3389/fmicb.2024.1402921 (PMC11097776; doi:10.3389/fmicb.2024.1402921)
Supplement: Supplementary file 1 [file Data_Sheet_1.docx]

Supplementary Material

**Table S1 First Round PCR Reaction System for Endophyte 16S rDNA**

| **PCR Reaction Components** | **PCR Reaction Volume** |
| --- | --- |
| Template DNA | 50 ng |
| KU63f | 0.5μL |
| KU1494r | 0.5μL |
| LNA-Mit63 | 1.5μL |
| LNA-Mit1492 | 1.5μL |
| LNA-Pla63S | 0.5μL |
| LNA-Pla1492S | 0.5μL |
| 2X Phanta Max master mix | 12.5μL |
| Add ddH_2_O to | 25μL |

**Table S2 First Round PCR Reaction Conditions for Endophyte 16S rDNA**

| **PCR Reaction Temperature** | **PCR Reaction Time** | **Cycling** |
| --- | --- | --- |
| 94℃ | 3min |  |
| 94℃ | 1min |  |
| 70℃ | 1min | 30cycles |
| 54℃ | 2min |  |
| 72℃ | 5min |  |
| 4℃ | ∞ |  |

**Table S3 Second Round PCR Reaction System for Endophyte 16S rDNA**

| **PCR Reaction Components** | **PCR Reaction Volume** |
| --- | --- |
| Template DNA  （The first round of PCR products） | 7.5μL |
| Forward Primer（1μM） | 2.5μL |
| Reverse Primer（1μM） | 2.5μL |
| 2X Phanta Max master mix | 12.5μL |
| Add ddH_2_O to | 25μL |

**Table S4 Second Round PCR Reaction Conditions for Endophyte 16S rDNA**

| **PCR Reaction Temperature** | **PCR Reaction Time** | **Cycling** |
| --- | --- | --- |
| 98℃ | 1min |  |
| 98℃ | 10s |  |
| 52℃ | 30s | 10cycles |
| 72℃ | 30s |  |
| 72℃ | 10min |  |
| 4℃ | ∞ |  |

**Table S5 Primers Used for Endophyte 16S rDNA** (Ikenaga et al., 2014)

| **PCR Reaction Components** | **PCR Reaction Volume** |
| --- | --- |
| KU63f | GCYTWAYACATGCAAGTC |
| KU1494r | GGYTACCTTGTTACGAC |
| LNA-Mit63 | GTCGAACGTTGTTTTCGG |
| LNA-Mit1492 | CTTCACCCCAGTCGAAGA |
| LNA-Pla63S | TCGGACGGGAAGTGGT |
| LNA-Pla1492S | CTTCACTCCAGTCACTAGC |
| Forward Primer（341F） | CCTACGGGNGGCWGCAG |
| Reverse Primer（805R） | GACTACHVGGGTATCTAATCC |

**Tab.S6** **First Round PCR Reaction System for Endophyte ITS**

| **PCR Reaction Components** | **PCR Reaction Volume** |
| --- | --- |
| Template DNA | 50 ng |
| Forward Primer（1uM） | 2.5μL |
| Reverse Primer（1uM） | 2.5μL |
| 2X Phanta Max master mix | 12.5μL |
| Add ddH_2_O to | 25μL |

**Table S7 First Round PCR Reaction Conditions for Endophyte ITS**

| **PCR Reaction Temperature** | **PCR Reaction Time** | **Cycling** |
| --- | --- | --- |
| 94℃ | 5min |  |
| 94℃ | 1min |  |
| 50℃ | 50s | 20cycles |
| 68℃ | 1min |  |
| 68℃ | 10min |  |
| 4℃ | ∞ |  |

**Table S8 Second Round PCR Reaction System for Endophyte ITS**

| **PCR Reaction Components** | **PCR Reaction Volume** |
| --- | --- |
| Template DNA  (The first round of PCR products) | 50ng |
| Forward Primer(1uM) | 2.5ul |
| Reverse Primer(1uM) | 2.5ul |
| 2X Phanta Max master mix | 12.5ul |
| Add ddH2O to | 25ul |

**Table S9 Second Round PCR Reaction Conditions for Endophyte ITS**

| **PCR Reaction Temperature** | **PCR Reaction Time** | **Cycling** |
| --- | --- | --- |
| 94℃ | 1min |  |
| 94℃ | 10s |  |
| 50℃ | 30s | 19cycles |
| 72℃ | 45s |  |
| 72℃ | 10min |  |
| 4℃ | ∞ |  |

**Table S10 Primers Used for Endophyte ITS** (Yao et al., 2019)

| **Amplified Fragments** | **Primers** | **Primer Sequences** |
| --- | --- | --- |
| First Round | ITS1F | 5'-CTTGGTCATTTAGAGGAAGTAA-3’ |
|  | ITS4 | 5'-TCCTCCGCTTATTGATATGC-3’ |
| Second Round | fITS7 | 5'-GTGARTCATCGAATCTTTG-3' |
|  | ITS4 | 5'-TCCTCCGCTTATTGATATGC-3' |

Ikenaga, M., & Sakai, M. (2014). Application of Locked Nucleic Acid (LNA) Oligonucleotide–PCR Clamping Technique to Selectively PCR Amplify the SSU rRNA Genes of Bacteria in Investigating the Plant-Associated Community Structures. *Microbes and Environments*, *29*(3), 286–295. https://doi.org/10.1264/jsme2.ME14061

Yao, H., Sun, X., He, C., Maitra, P., Li, X.-C., & Guo, L.-D. (2019). Phyllosphere epiphytic and endophytic fungal community and network structures differ in a tropical mangrove ecosystem. *Microbiome*, *7*(1), 57. https://doi.org/10.1186/s40168-019-0671-0


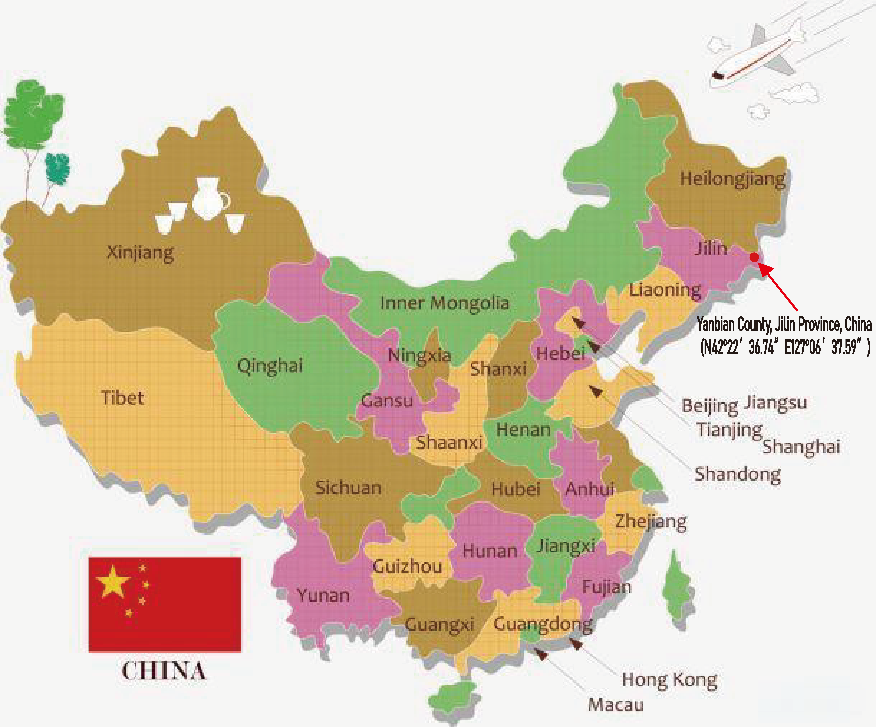


**Figure s1 Sampling sites**
